# Organic and Ionic Liquids Electrolyte Solutions as Versatile Media for Metallic Lithium Recovery

**DOI:** 10.3390/ma18122899

**Published:** 2025-06-19

**Authors:** Mihai Tudor Olaru, Alexandru Matei, Irina Atkinson, Adelina Ionela Matei, Elena Bacalum, Miruna Iota, Ana-Maria Popescu

**Affiliations:** 1 National Research & Development Institute for Non-Ferrous and Rare Metals-IMNR, 178-184 Blv. Biruinței, Pantelimon 077145, Romania; o.mihai@imnr.ro (M.T.O.); alex.matei@imnr.ro (A.M.); amatei@imnr.ro (A.I.M.); elena.bacalum@imnr.ro (E.B.); iota.miruna@imnr.ro (M.I.); 2 Ilie Murgulescu “Institute of Physical Chemistry”, 202 Spl. Independenței, Bucharest 060021, Romania; irinaatkinson@yahoo.com

**Keywords:** lithium electrodeposition, organic/ionic liquid electrolytes, cyclic voltammetry, XRD, SEM, chemical analysis/FTIR

## Abstract

For various applications, particularly in battery technology, there is a significant demand for uniform, high-quality lithium or lithium-coated materials. The use of electrodeposition techniques to obtain such materials has not proven practical or economical due to the low solubility of most lithium salts in suitable solvents. In this study, we propose efficient lithium electrodeposition processes and baths that can be operated at low temperatures and relatively low costs. We utilized organic solvents such as dimethyl acetamide (DMA), dimethylforamide (DMF), and dimethyl sulfoxide (DMSO), as well as a mixture of DMSO and ionic liquid [1-Butyl-3-methylimidazolium bis(trifluoromethylsulfonyl)imide BMIMTFSI]. Lithium salts such as LiCl, Li_2_CO_3_, and LiNO_3_ were tested. Lithium metal was deposited on copper substrates at different temperatures and selected current densities within an argon-filled glovebox using a DC power source or a PARSTAT-4000A potentiostat. Cyclic voltammetry (CV) was employed to determine and compare the deposition processes. The obtained deposits were analyzed through visual inspection (photography) and scanning electron microscopy (SEM). Chemical analysis (ICP-OES) and XRD confirmed the presence of lithium and occasionally lithium hydroxide in the deposits. The best results were achieved with the deposition of lithium from DMSO-LiNO_3_ and DMSO-BMIMTFSI-LiNO_3_ systems.

## 1. Introduction

The global transition to renewable energy and decarbonized transportation [1] has generated an extraordinary demand for lithium, raising concerns regarding the eventual depletion of global sources of lithium and the impact on the environment of traditional extraction methods [2].

Lithium (Li), the lightest metal and a key element in modern technology, has seen an increase in demand due to its important role in energy storage, especially in rechargeable lithium-ion batteries (LIBs). There is a substantial consumer market for lithium-ion batteries (LIBs), from electric vehicles (EVs) and portable electronics to grid energy storage systems [3]. In comparison to traditional lead–acid and dry batteries, lithium-ion batteries (LIBs) demonstrate advantages such as higher output voltage, superior energy density, and excellent cycle performance.

Current industrial methods for lithium recovery and recycling, such as pyrometallurgy, hydrometallurgy, and especially molten salt technology (MST), are energy-intensive and involve the use of toxic chemicals, raising significant environmental concerns. In this context, electrochemical methods, in particular electrodeposition, offer a more sustainable and cost-effective alternative. Emerging electrochemical methods for lithium recovery, such as electrolysis, electrodialysis, and capacitive deionization (CDI), offer promising alternatives. These techniques are characterized by their high lithium selectivity, rapid ion capture, and low energy consumption [3]. Recovering metallic lithium using organic solvents and ionic liquids (ILs) is a growing area of research, especially due to the increasing need to recycle lithium from batteries or industrial waste. A recent review presents the promising iono-metallurgy for recovering metals from spent batteries [4]. All the electrodepositions of metallic lithium from the studied electrolytes represent an area of interest for lithium-based battery technology and other electrochemical applications. This process involves reducing lithium ions (Li^+^) in the electrolyte to deposit metallic lithium on a conductive substrate. However, one of the persistent challenges for these electrochemical processes is the low solubility of lithium salts in conventional solvents, which can limit the efficiency and scalability of lithium recovery through electrodeposition.

Another major issue in lithium electrodeposition is obtaining a uniform lithium deposit, as demonstrated by the formation of lithium dendrites. During lithium deposition, the Li^+^ ions tend to accumulate unevenly on the surface of the electrode, forming structures similar to needles, known as dendrites. The growth of dendrites leads to the repeated formation and rupture of the solid electrolyte interphase (SEI) film, consuming both the lithium metal and electrolyte, which significantly reduces the life of the battery and its efficiency [5]. Factors such as the structure of Li^+^ ion solvation in the electrolyte, the process of desolvation, and the SEI properties influence the lithium deposits. Various methods have been investigated to increase the interfacial stability of the Li electrode, such as the use of various electrolyte solvents, salts, and additions [6]. It has been shown that electrolyte additives such as LiNO_3_ change the solvation structure [7], which can reduce the growth of dendrites by promoting a more uniform flow of lithium ions.

In the context of lithium recovery, including electrodeposition, preventing dendrites is equally important. Unfortunately, even though there are many studies on lithium batteries, there are few or none on the electrodeposition of lithium on different supports for being used in the recycling of batteries. For efficient lithium electrodeposition, especially when using organic solvents, such as dimethyl acetamide (DMA), dimethyl formamide (DMF), and dimethyl sulfoxide (DMSO) (as used in this study), the electrolyte environment significantly influences lithium ion deposition. By optimizing the electrolyte and solvents used in the deposition process, it is possible to reduce the formation of dendrites, thus improving both the efficiency and the safety of lithium recovery by electrochemical methods.

In response to this challenge, recent research has focused on developing more efficient lithium electrodeposition techniques that can operate at lower temperatures and costs. Organic solvents, such as DMA, DMF, and DMSO, have shown potential for increasing the solubility of lithium salts and improving the overall deposition process, and conducting to fine grains and smooth lithium deposits [8]. This study proved that the addition of water to a LiNO_3_-DMF solution increases the current efficiency of lithium deposition. Another study investigated the effect of H_2_O addition to electrolytes on only metal electrodeposition. It was found that trace amounts (25–50 ppm) of H_2_O can be an effective additive in LiPF6-based electrolytes to suppress Li dendrite growth during Li deposition [6]. The degradation of lithium-ion batteries (LiBs) during operation represents a complex and critical challenge, primarily driven by various electrochemical side reactions occurring across all battery components. Among these mechanisms, lithium plating on the anode stands out as a significant contributor to performance deterioration. Accurately predicting the onset and extent of lithium plating further compounds the difficulty due to the intricate interplay of material properties, operational conditions, and electrochemical dynamics. The lithium plating mechanism has not yet been elucidated. In order to avoid LiB degradation, newer studies reviewed the current literature on lithium plating and on current electrochemical models for lithium plating [9].

As we could not find any in-depth study of lithium electrodeposition on various supports from organic electrolytes, we supposed that it would be a good idea to make one. These organic solvent systems offer the advantage of reducing the temperature and energy requirements of the deposition process while maintaining high deposition quality. Additionally, we believed that the use of ILs, such as 1-butyl-3-methylimidazolium bis(trifluoromethylsulfonyl)imide (BMIMTFSI), in combination with DMSO, could improve lithium deposition.

In this study, we investigate the electrodeposition of lithium using a variety of lithium salts, including LiCl, Li_2_CO_3,_ and LiNO_3_, in different organic/IL solvent systems. Specifically, we utilize solvents such as DMA, DMF, and DMSO, as well as a mixture of DMSO with BMIMTFSI, to evaluate their effectiveness in improving the deposition of lithium on copper substrates. The electrodeposition processes are analyzed through cyclic voltammetry (CV), and the resulting deposits are characterized by different techniques.

## 2. Materials and Methods

**Figure 1:**
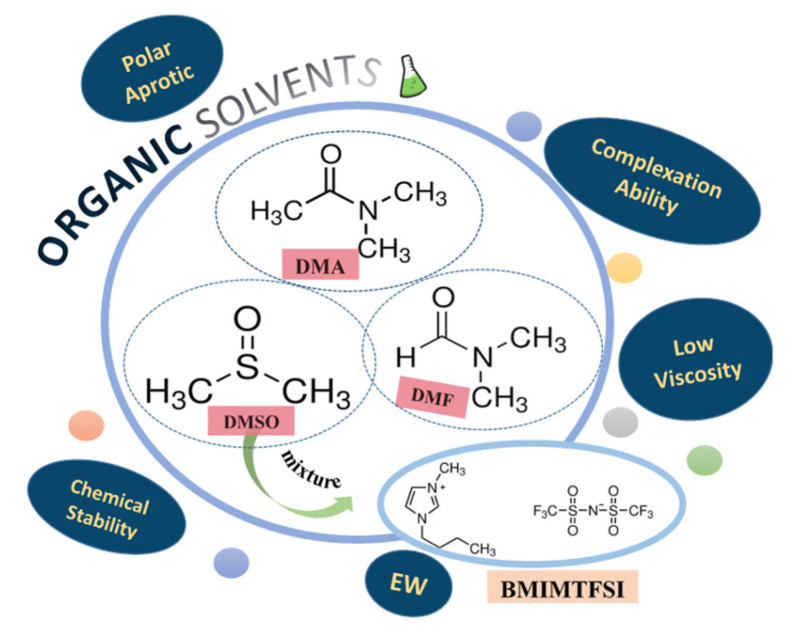
Organic and organic ionic liquid solvents used for the lithium electrodeposition.

Taking into account the literature, we decided to use an organic or organic ionic liquid electrolyte for lithium electrodeposition. All used materials were purchased from Merck (Darmstadt, Germany) or Alpha Aesar (Ward Hill, MA, USA) and of purity >95%. We utilized as an electrolyte the following solvents (Figure 1): dimethyl acetamide (DMA), dimethylforamide (DMF), and dimethyl sulfoxide (DMSO), as well as a mixture of DMSO with an ionic liquid [1-Butyl-3-methylimidazolium bis(trifluoromethylsulfonyl)imide BMIMTFSI]. As for lithium salts, we used lithium chloride (LiCl), lithium carbonate (Li_2_CO_3_), and lithium nitrate (LiNO_3_). These electrolytes were chosen due to their beneficial properties: polar aprotic solvents with good chemical stability, low viscosity, and large electrochemical windows (EWs) and complexing abilities.

The CV experiments were carried out with a potentiostat/Galvanostat PARSTAT 4000A (Princeton Applied Research, Oak Ridge, TN, USA), equipped with Versa Studio software v2.66.2. The used electrochemical cell was a three-electrode setup (WE = copper plate; CE = QRE= Platinum plate). Before each experiment, the electrodes were cleaned with abrasive paper of various sizes, polished with alumina paste, washed with bi-distilled water and absolute ethyl alcohol, then washed with acetone several times, then dried well, and then washed with the electrolyte used in the experiment. The copper electrodes were masked with insulating tape, leaving an exposed surface area of 2.0 cm^2^. They were dried and immediately transferred to an argon-filled glove box (MB200-MBROWN, Garching, Germany, <1 ppm water and oxygen content) for cell assembly.

**Figure 2:**
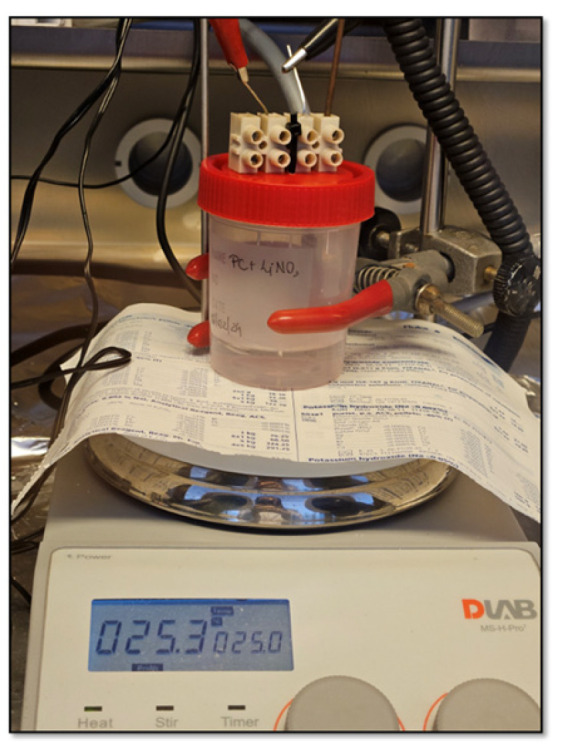
Electrodeposition cell.

Lithium metal was deposited onto copper substrates at room temperature within an argon-filled glovebox, using either a power source (DC Dual Supply 6145-PeakTech, Ahrensburg, Germany) or a PARSTAT-4000A potentiostat (Princeton Applied Research, Oak Ridge, TN, USA). The cell used was a little one, as in Figure 2. Copper is commonly used as a substrate for lithium electrodeposition because of its good conductivity and high surface area. Copper does not react easily with lithium, but careful control of the deposition parameters is required.

The samples of copper substrate with the electrolytic deposits obtained in dry boxes were prepared for further analysis. Samples were purged with an Ar stream to remove as much electrolyte as possible and then were placed in hermetically sealed glass vials filled with argon. We chose this option to eliminate as much as possible the possibility of contamination of the deposits with oxygen, although during the analysis experiments, the samples were kept in air, but an attempt was made to minimize this time. We tried to keep the samples in tightly closed glass cells filled with paraffin oil of high purity, but the lithium deposit was oxidized.

The obtained deposits were analyzed through visual inspection (photography), scanning electron microscopy (SEM) FEI QUANTA 250 (FEI Company, Eindhoven, The Netherlands), chemical analysis (ICP-OES) model 725 (Agilent Techn., Santa Clara, CA, USA), and an X-ray diffraction (XRD) Ultima IV diffractometer (Rigaku, Tokyo, Japan). Photographic images were taken with a NIKON digital camera (Nikon, Tokyo, Japan).

A SEM analysis was performed using the secondary electron detector (ETD) (FEI Company, Eindhoven, The Netherlands) and the secondary backscattered electron detector (ABS) (FEI Company, Eindhoven, The Netherlands). The microscope is integrated with the energy dispersive X-ray spectrometer, manufactured by EDAX (Mahwah, NJ, USA), consisting of ELEMENT Silicon Drift Detector Fixed (EDAX, Mahwah, NJ, USA), Element EDS Analysis Software Suite (APEX™ 1.0). Unfortunately, Li cannot be detected by EDS.

The XRD patterns were recorded with a diffractometer equipped with parallel beam optics and a thin-film attachment using Cu Kα radiation (λ = 1.5405 Å, operated at 30 mA and 40 kV, over the 2θ range 10–100°, at a scanning rate of 2°/min, with a step width of 0.02°).

Unfortunately, for any of the analysis experiments, we could not make/build a special concept to protect the sample from atmospheric contamination.

The FT-IR measurements using an ABB-MB3000 Fourier Transform Infrared Analysis System (ABBAsea Brown Boveri Ltd., Zurich, Switzerland) were used to characterize the DMSO-LiNO_3_ electrolyte before and after electrolysis.

## 3. Results and Discussions

### 3.1. Study of Lithium Salts Dissolution in the Chosen Solvents

In order to choose the best electrolytes for the electrodeposition of lithium, intermediate studies of the solubilization of some lithium salts in various organic solvents and organic/ionic liquids were carried out. Table 1 summarizes these studies and the results obtained. After this study, we concluded that the most easily soluble lithium salt was lithium nitrate, which we used in the electrodeposition studies in different environments.

### 3.2. Cyclic Voltammetry of the Lithium Electrodeposition

In order to determine the deposition potentials of lithium in the electrolytes under study, we performed a preliminary cyclic voltammetry (CV) study. The CVs were performed at different scan rates (sc = 0.025–0.2 V/s) and at 298 K for the electrolytes totally dissolved from Table 1. The electrodeposition processes were evaluated according to the CVs criteria [10].

**Figure 3:**
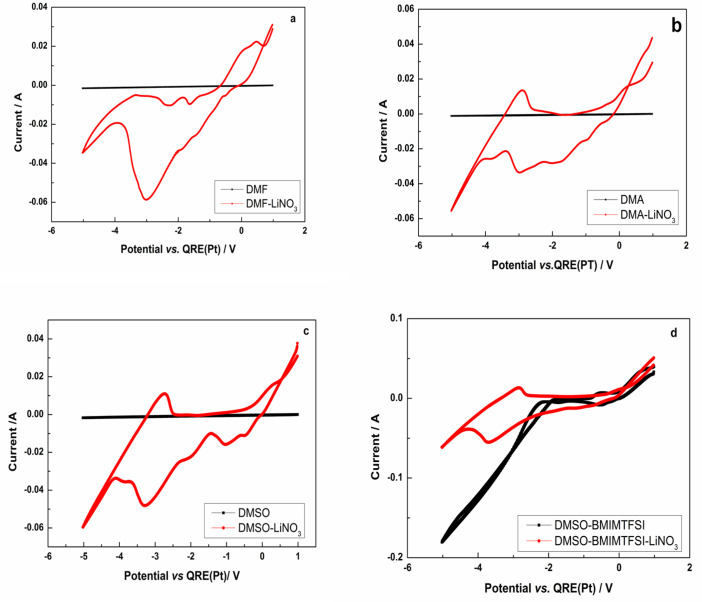
Cyclic voltammograms of backgrounds and lithium deposition for sc = 0.2 V/s: (**a**) DMF/DMF-LiNO_3_; (**b**) DMA/DMA-LiNO_3_; (**c**) DMSO/DMSO-LiNO_3_; (**d**) DMSO-BMIMTFSI/DMSO-BMIMTFSI-LiNO_3_.

First, we perform the CVs of the background (the solvent/black line) and then the CVs with the addition of LiNO_3_ (the red line). This allowed us to demonstrate through the superposition of CVs (at the same scan rate, 0.2 V/s) that the background does not interact with the lithium deposition (Figure 3). The same behavior is observed for all scanning speeds used in the study.

For the DMA-LiNO_3_ system (Figure 3a), DMA only (black) shows almost no significant cathodic activity until very negative potentials, while DMA–LiNO_3_ (red) shows a distinct cathodic peak around −3.5 V to −4.5 V, indicating a reduction reaction, corresponding to the electrochemical reduction of LiNO_3_. LiNO_3_ is known to reduce at the lithium surface, forming species like LiNxOy, Li_2_O, and possibly Li_3_. The cathodic peak appears only after 0.10 V/s, and the position of this peak is shifted toward more negative values with increasing scanning speed, reaching the value of −3.815 V at 0.2 V/s. The rest of the cathodic peaks correspond to the background (DMA). This deposition process was found to be a quasi-reversible one. The anodic (oxidative) sweep shows a broad rise in current in the DMA-LiNO_3_ sample starting around 0 V up to 1.5 V, which is significantly higher than in pure DMA. This could be due to the oxidation of previously formed components or enhanced solvent oxidation facilitated by changes in surface chemistry due to LiNO_3_. The increase in the oxidative current suggests a more reactive surface—possibly due to the products from the LiNO_3_ reduction. The hysteresis between cathodic and anodic sweeps in DMA-LiNO_3_ indicates irreversible redox processes, likely associated with SEI formation.

For the DMF-LiNO_3_ system (Figure 3b), DMF is relatively stable but will reduce at low potentials and can decompose anodically. Large cathodic peak near –2.8 to –3.0 V: this is likely the reduction of Li^+^ to Li^0^ (lithium plating) or DMF solvent reduction. Since this peak is not present in pure DMF, it is strongly associated with LiNO_3_ addition, likely Li^+^ reduction, and could also be competitive nitrate reduction, which can overlap in this range. On the anodic region, multiple small anodic waves and a significant rise around 1.5–2.0 V are observed. These may correspond to the oxidation of reduced nitrate species, lithium stripping, or oxidative decomposition of reaction products. The increase (the red line) in current toward more positive potentials indicates electrolyte oxidation. The lithium reduction at 0.2 V/s takes place at −3.011 V, and the process is irreversible, suggesting electrolyte decomposition and the formation of passivation layers.

From Figure 3c, we found that for the DMSO-LiNO_3_ system, DMSO has a wide electrochemical window in the region scanned, and it is electrochemically stable in the studied range. On the baseline (DMSO alone—the black line),the current is nearly flat across the potential range, which indicates that no significant redox reactions occur in pure DMSO under these conditions. On the DMSO-LiNO_3_ (the red line), there are several distinct redox peaks visible and corresponding to electrochemical reactions induced by the presence of LiNO_3_ in DMSO, suggesting reduction processes at more negative potentials and oxidation processes at more positive potentials. On the cathodic region of the voltammogram, we observed multiple cathodic peaks in the region −1 V to −4.5 V. The peaks from −0.5 V to −2.0 V were assumed to correspond to the (NO_3_)^−^. Possible DMSO reduction takes place at lower potentials (−2.0 V). Around −2.5 V to −3.5 V, it is likely reduction of nitrate (NO_3_^−^) to nitrite (NO_2_^−^) or other reduced nitrogen species (NO, NH_3_/N_2_O). Around −4.0 V to −4.5 V, further reduction or solvent breakdown (i.e., DMSO reduction to DMS or NO_3_ irreversible decomposition to NO_2_) is possible. There is a sharp increase in cathodic current beginning around −3.5 V, which peaks near −4.0 V to −4.5 V. This region most likely includes lithium ion reduction (Li^+^ → Li^0^) and possibly overlaps with solvent reduction (DMSO) or nitrate reduction byproducts. Therefore, the reduction of lithium most likely begins just before −3.5 V and becomes significant near −4.0 V to −4.5 V vs. Pt QRE, possibly overlapping with solvent reduction (DMSO) or nitrate reduction byproducts. At the same time, the high current suggests a faradaic process (not simple adsorption). No clear stripping peak (the oxidation of Li^0^ back to Li^+^) appears on the anodic scan, suggesting irreversible lithium plating or that lithium is passivated. The lithium reduction process appears to be a diffusion-limited, irreversible electrodeposition process. Here is a breakdown of the characteristics that support this conclusion: an electrochemical reduction of solvated lithium ions to metallic lithium, typically resulting in plating onto the electrode surface; a sharp cathodic increase indicates a nucleation and growth process. This is typical for metal plating/deposition, the sharpness suggesting nucleation and the growth of lithium metal on the electrode. We can assume that for the DMSO-LiNO_3_ system, the process of lithium electrodeposition at a 0.2 V/s scan rate begins at −4.266 V.

**Figure 4:**
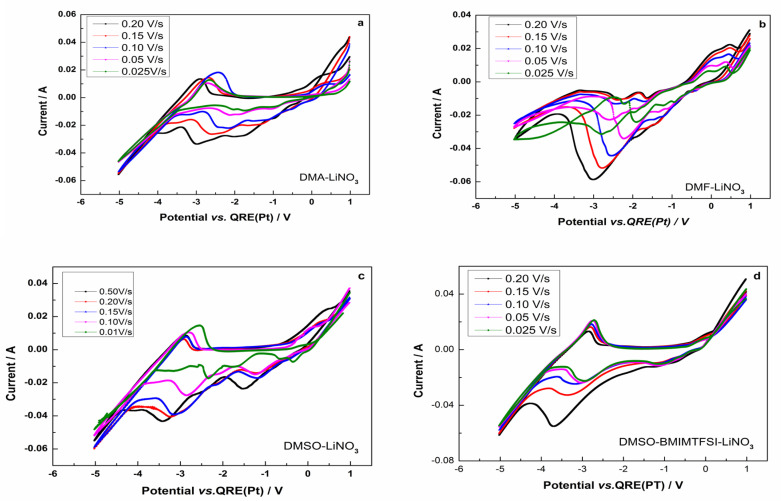
Cyclic voltammograms obtained during the electrodeposition of lithium from LiNO_3_ at 25 °C in different electrolytes and with different scan rates: (**a**) DMA; (**b**) DMF; (**c**) DMSO; (**d**) DMS-BMIMTFSI.

For the DMSO-BMIMTFSI-LiNO_3_ (Figure 4d), the addition of LiNO_3_ (the red curve) significantly affects the electrochemical behavior, altering both the cathodic and anodic currents and indicating changes in electrochemical processes, possibly due to Li^+^ interaction with the electrode or electrolyte species. On the cathodic region, the red curve shows increased current response at negative potentials (around −4 V to −2 V), suggesting enhanced reduction processes or new reduction reactions due to the presence of LiNO_3,_ while the black curve is more stable and linear in this region, suggesting fewer or less intense redox processes. On the anodic region (positive potential), both curves show oxidation processes, but the red curve (with LiNO_3_) has a broader and possibly more intense response, indicating modified oxidation pathways. The electrochemical window appears to be wide in both cases, but the red curve (LiNO_3_) shows more defined redox features, possibly indicating a narrower stable window but with more electrochemical activity. The addition of LiNO_3_ introduces new redox-active species or changes the double-layer structure and ion interactions, influencing the electron transfer kinetics. The DMSO–BMIMTFSI system alone is more inert, while the addition of LiNO_3_ activates additional processes, likely involving lithium deposition or interfacial reactions. Around −3.5 to −4.5 V vs. QRE, you see a pronounced cathodic peak (in the red curve only). This peak does not appear in the black trace (which lacks LiNO_3_ and therefore lithium ions), confirming it is associated with lithium. Around −3.5 to −4.5 V vs. QRE, you see a pronounced cathodic peak (in the red curve only). This peak does not appear in the black trace (which lacks LiNO_3_ and therefore lithium ions), confirming it is associated with lithium. The lithium deposition peak is most likely the sharp cathodic feature in the red curve around −45.495 V vs. QRE (Pt) for 0.2 V/s. The characteristic of lithium deposition in this case is nucleation and growth (lithium nucleates as small metallic clusters on the electrode surface and these clusters grow into a metallic layer); irreversible or quasi-reversible process (shows a sharp cathodic peak and a separate anodic (stripping) peak on the reverse scan); and high overpotential (requires significant negative overpotential to initiate the nucleation process).

The variation in the cyclic voltammograms of all used electrolytes, with the scan rates, is presented in Figure 4. One can observe from Figure 4 that the size of the peaks from the CVs increases with increasing scanning speed. On all voltammograms, a strong increase in current is observed after zero volts, which indicates an anodic gas evolution (we assume this is oxygen or nitrogen compounds).

Table 2 summarizes the values of the deposition potentials of metallic lithium in the studied electrolytes at a 0.2 V/s scan rate.

### 3.3. Electrodeposition of Metallic Lithium

The electrodeposition of lithium on copper substrates was performed on the electrolytes formed by a solvent (organic DMA, DMF, DMSO, or organic/ionic liquid DMSO + BMIMTFSI) and a solute (lithium salt). For all the systems, we conducted the deposition at a moderate temperature, often around 298 K, to maintain electrolyte stability. The electrodeposition of metallic lithium from these electrolyte systems requires careful control of the conditions because lithium is highly reactive, especially in organic solvents.

#### 3.3.1. System DMA-LiNO_3_

**Figure 5:**
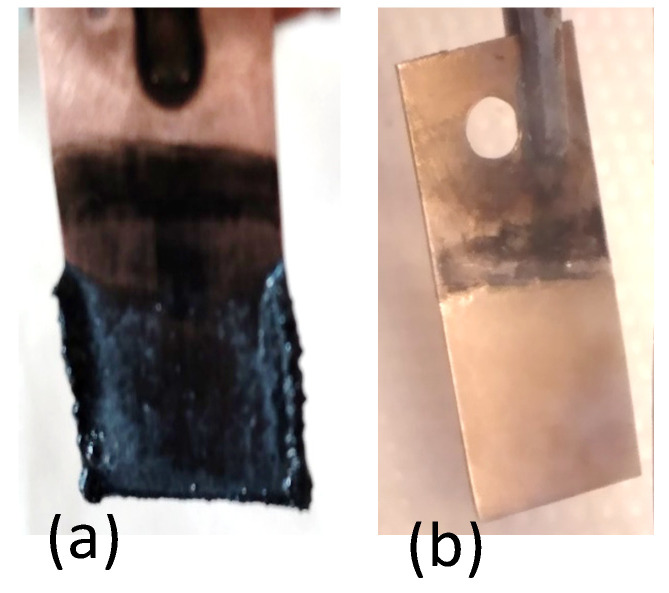
Photos of the obtained lithium deposits on copper substrate from the electrolyte DMA-LiNO_3_ in different conditions: (**a**) 3.5 V, 0.1 mA; (**b**) 4 V, 0.01 A.

DMA is a polar aprotic solvent with a high dielectric constant, stabilizing Li^+^ ions in the solution. The concentration of LiNO_3_ used in the tests was between 0.6 and 0.7 M. Using 0.7 M LiNO_3_ and making the electrolysis at 298 K for more than 4 h at 4.0 V and 0.1 A, we succeeded in obtaining a black and dense deposit (Figure 5a). From the thick deposit on Cu, particles are detached and fall in to the electrolyte. At the end of the electrolysis process, the color of the electrolyte changes to a yellow-green phenomenon that may be due to the formation of chemical species or degradation products generated during the electrochemical reaction. During electrolysis, especially at the anode, nitrate ions (NO_3_^−^) can be oxidized, potentially forming nitrogen oxides (NO, NO_2_, and N_2_O_4_). These compounds can have characteristic colors, with NO_2_ being particularly yellow-brown to yellow-green. The exact shade might vary depending on the specific reaction conditions and concentration.

DMA itself could also undergo oxidation or reduction during electrolysis, producing products that may have color. There might be side reactions involving the solvent (DMA) or LiNO_3_, especially at the electrodes, leading to the formation of colored compounds (e.g., amine oxidation, which can be yellow or green). These can include metal complex formation (e.g., if the lithium ion interacts with other electrolytic products) or other unstable intermediates that impart color.

When the working temperature is lowered, a washout occurs at the cathode. The same thing happens if the voltage or the current density is increased to 7 V. This is due to a much higherH_2_ evolution. By changing the working conditions to 6 V and 0.02 A, no deposit on the Cu was observed, but the electrolyte turned blue-green.

The electrolysis process may cause a change in the solvent environment, which could result in the formation of colored species. The combination of lithium ions, nitrate ions, and DMA can lead to various electrochemical products, contributing to the color change. We assumed that during electrolysis, metal ions can be reduced or oxidized, leading to the formation of colored metal complexes. For instance, if there are transition metal impurities or electrodes (like copper, iron, or cobalt), they could interact with the DMA or nitrate ions, forming colored complexes that may exhibit blue or green hues.

In summary, the blue-green color observed after electrolysis of DMA-LiNO_3_ is likely due to the formation of colored electrochemical products or complexes resulting from the interaction between metal ions, the electrolyte, or the electrolysis products.

The best results were obtained by performing the electrodeposition at 3.2 V and 0.01 A, for 6 h, with a uniform and adherent gray deposit (Figure 5b). This result is in accordance with the CV of DMA-LiNO_3_. Also, a lower current density and a longer time generally produce a better-quality deposit in electrolysis.

#### 3.3.2. System DMF-LiNO_3_

For the system DMF-LiNO_3_, the concentration of LiNO_3_ was found to be optimized between 0.1 M and 1.0 M, depending on the specific setup. We used the same 0.7 M LiNO_3_ concentration as in the case of the other system tested.

**Figure 6:**
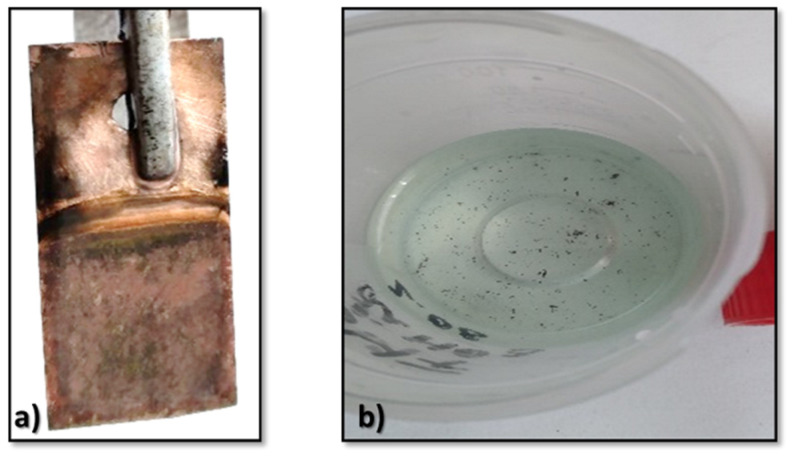
The deposit (**a**) and the fine deposit found in the electrolyte (**b**) during electrolysis of DMF-LiNO_3_.

After performing electrolysis for 5 h at 4 V and 0.01 A, IMNR obtained a very fine brown, dark, and thin deposit (Figure 6a) not detected by XRD. We believe that the thin and black-to-brown appearance of the deposit likely arises from the combination of carbonaceous residues from DMF decomposition, metal or metal oxide content (an ICP-OES analysis showed the presence of lithium and oxygen in the deposit obtained), and the thin-film nature itself. After electrolysis, small black particles were floating in the electrolyte. The results are presented in Figure 6b.

#### 3.3.3. System DMSO-LiNO_3_

**Figure 7:**
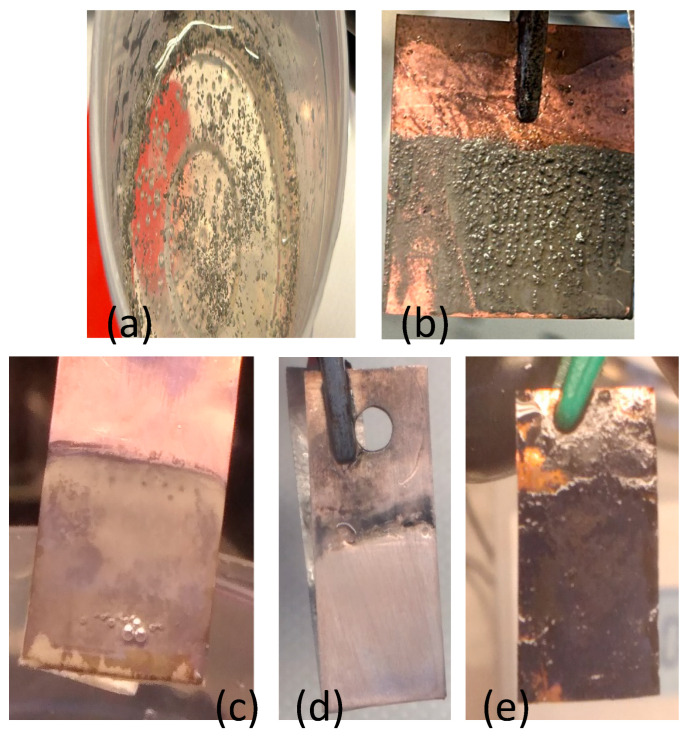
DMSO-LiNO_3_ electrolysis: (**a**) deposited particles fallen into the electrolyte; (**b**) gray granulated deposit; (**c**) metallic lithium bubbles; (**d**) smooth gray lithium; (**e**) deposit from DMSO-BMIMTFSI-LiNO_3_.

The aprotic solvent, DMSO, is often chosen because it can dissolve lithium salts like LiNO_3_ efficiently and allows lithium deposition without the risk of solvate formation that would occur in other solvents like water. While DMSO is a good and stable solvent for lithium salts, it must be handled carefully, as it can degrade under certain electrochemical conditions, especially at high voltages or in the presence of reactive species. DMSO can undergo electrochemical reduction at the cathode, where it may be reduced to form dimethyl sulfide (DMS) or other sulfur-containing byproducts [11]. At the same time, at the anode, lithium nitrate (LiNO_3_) could undergo oxidation, which may produce nitrogen oxides (such as NO_2_) and potentially lithium oxide (Li_2_O). Lithium oxide can appear as black particles, especially if it is formed in an uncontrolled manner or with excessive current. By preliminary experiments, we found that the concentration of LiNO_3_ should be optimized, typically between 0.5 M and 2 M, depending on the specific deposition conditions and desired current densities. Good results were obtained for a concentration of 0.7 M LiNO_3_, which is in good correlation with the other systems already tested. In some cases, dark gray particles containing lithium species may float on the surface of the electrolyte (Figure 7a). So, performing the electrodeposition at 298 K and 4.13 V/0.02 A, we obtained a gray granulated deposit (Figure 7b) for which the chemical analysis proved to be Li.

During some experiments (at higher voltages, 6–7 V), we observed that, especially at the cathode, foaming is present and gas bubbles come out. We assumed that foaming at the cathode in this electrolysis is largely due to the evolution of hydrogen gas (if water is present, which can happen in traces in many electrochemical systems) and possibly some side reactions involving the electrolyte components themselves. These gas bubbles accumulate at the cathode surface and create the foaming effect. Also, another possibility for this foaming could be that DMSO also has some tendency to interact with lithium ions and may form complexes, which can facilitate the release of gases like hydrogen or possibly even gases from side reactions involving DMSO degradation [DMS, sulfur dioxide (SO_2_), or other volatile products]. It is also considered that if the applied potential is too high, side reactions such as the reduction of other species present in the electrolyte could occur, resulting in gas formation (for example, LiNO_3_ could decompose under high voltage, leading to the production of nitrogen oxides and other byproducts).

**Figure 8:**
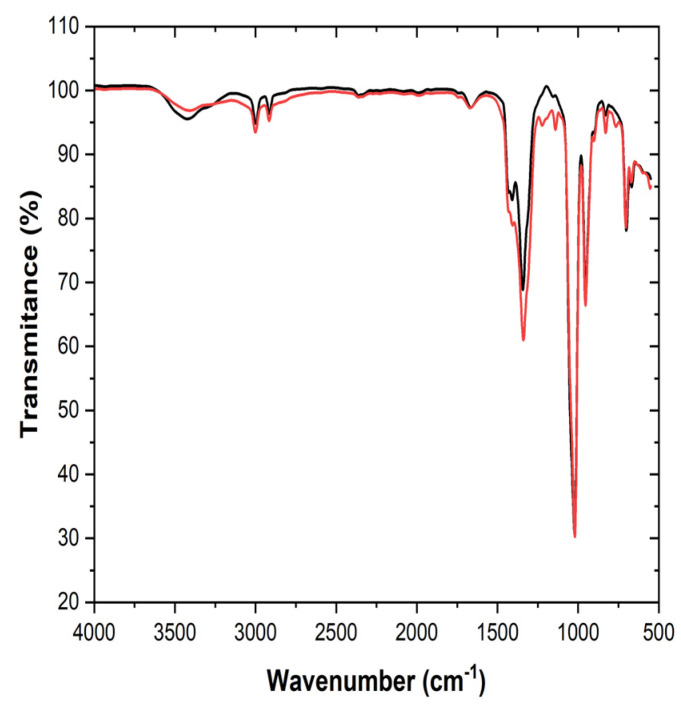
FT-IR spectra of the DMSO-LiNO_3_ electrolyte before (black) and after (red) the lithium electrodeposition.

To support these claims, we performed FT-IR experiments on the DMSO-LiNO_3_ electrolyte before and after electrodeposition (Figure 8).

The identification of specific groups before deposition gave the following results: the band in the 3421 cm^−1^ region is specific to O-H stretching vibrations (considering that the sample is a solution), and the bands in the 2999 cm^−1^ and 2914 cm^−1^ region, respectively, are specific to C-H stretching bands, in which the band at 2999 cm^−1^ has an asymmetric C-H stretch from CH_3_, and the band at 2914 cm^−1^ has a symmetric C-H stretch also from CH_3_. These bands are characteristic of DMSO and represent the carbon–hydrogen bonds in the methyl groups; the band in the 1664 cm^−1^ region is assigned to the stretching vibrations of the C=O or C=N bonds. In DMSO, this band is not characteristic but could be related to interactions with LiNO_3_. Considering that the sample is a solution, it could be associated with the H-O-H deformation vibration; the bands in the region 1431 cm^−1^ and 1432 cm^−1^ areassociated with the NO_3_^−^ deformation vibration; and the band in the region 1022 cm^−1^ is assigned to the S=O stretching vibration (a DMSO characteristic). This is the main band for the sulfoxide group, very sensitive to chemical interactions; the band at 955 cm^−1^ is associated with the vibration of the molecular structure of DMSO or secondary bonds with NO_3_^−^. This band could reflect secondary interactions or molecular associations (for example, between DMSO and nitrate); the band at 702 cm^−1^ is most likely assigned to DMSO, reflecting a deformation vibration of the S=O group, but considering also the LiNO_3_ compound, it is possible to have an overlap of vibrations between the planar deformation of NO_3_^−^, influenced by the bonds with DMSO.

The identification of specific DMSO-LiNO_3_ groups in the sample after deposition: the band in the region of 3408 cm^−1^ is specific to O-H stretching vibrations if we consider that the sample is a solution. The slight shift to lower values (from 3421 to 3408 cm^−1^) suggests an increase in hydrogen bonds after deposition, but it is possible that the interaction with nitrate or lithium increased the polarization of water in the range of 3001 cm^−1^ and 2916 cm^−1^ assigned to the asymmetric and symmetric C-H stretching vibration of CH_3_ (DMSO). The bands remain relatively unchanged, which suggests that the methyl groups in DMSO are not significantly affected by the deposition process; the 1668 cm^−1^ band, which is less visible here, is associated with the stretching vibration of the C=O bond or H-O-H deformation; and in the range of the bands 1429 cm^−1^ and 1340 cm^−1^, it is a vibration deformation of the nitrate ion (NO_3_^−^).The extension of the range (now wider) and the changes in position indicate a reduction in the symmetry of the nitrate ion, probably due to a stronger interaction with DMSO, and the band at 1020 cm^−1^ is associated with the S=O stretching vibration of DMSO. The band remains very intense but has a slight shift to lower values (1022 → 1020 cm^−1^), suggesting a stronger coordination of the sulfoxide group (S=O) with the Li^+^ ion after deposition; the band at 955 cm^−1^ isassociated with the structural vibration of DMSO or secondary interactions with NO_3_^−^. The persistence of this band without major changes indicates the stability of the interactions between DMSO and the ions in the solution; the band at 704 cm^−1^ is attributed to the deformation vibration of the S=O or NO_3_^−^ group. The slight shift suggests subtle changes in symmetry. Identification of the species groups is in good agreement with the literature for DMSO [12,13,14].

The conclusion based on FTIR interpretations before and after deposition is the following:

1. Changes in ion–solvent interactions:
  - The shift in the characteristic bands for the S=O group in DMSO (1022 cm^−1^ → 1020 cm^−1^) and the high intensity of this band after deposition indicate a strong coordination between DMSO and lithium ions (Li^+^).
  - The broadening and modification of the bands assigned to nitrate (NO_3_^−^, 1429–1340 cm^−1^) suggest a reduction in the symmetry of the nitrate ion, most likely through interactions with DMSO or through the formation of new molecular structures.
2. Stability of DMSO:
  - The C-H bands (3001–2916 cm^−1^) remain unchanged, indicating that the methyl groups in DMSO are not chemically affected and are only involved in physical interactions.
3. Formation of post-deposition complexes:
  - The observed changes in the S=O and NO_3_^−^ bands suggest the formation of a DMSO-Li^+^-NO_3_^−^ complex, in which DMSO plays a ligand role and the nitrate becomes more asymmetric.

In conclusion, the FTIR spectrum indicates strong interactions between DMSO and LiNO_3_, especially through the bond between sulfoxide, oxygen and lithium, which leads to changes in the characteristic frequencies. The most relevant are the shift in the S=O band and changes in the nitrate bands, which confirm the complexity of the electrolyte.

If the conditions for lithium deposition (such as current density or temperature) are not optimal, lithium may not deposit in a uniform manner. Sometimes, during the electrodeposition of lithium (at 5.06 V and 0.9 mA) on the copper cathode, some lithium metal bubbles can appear (Figure 7c). In some cases, when there is trace water or impurities in the electrolyte (and DMSO is quite hygroscopic), the voltage applied during the electrodeposition can be high enough to reduce water (or other contaminants) at the cathode, resulting in the evolution of hydrogen gas. This can lead to the formation of gas on the surface of the cathode. Even though the primary reaction is the deposition of lithium metal, any side reactions (such as HER) can interfere and cause gas formation. The above-mentioned conditions could cause some areas of the cathode to evolve gases due to localized overpotentials, leading to lithium metal bubble formation. So, the metallic lithium produced can form small bubbles because of the local conditions at the cathode, such as hydrogen gas evolution or the growth of lithium metal from the reduction of lithium ions. This can lead to a bubbling effect as the newly formed lithium metal detaches from the cathode surface or as the reaction continues over time.

During our study, we found that this is beneficial and even necessary to perform pre-electrolysis at 2.5–3.5 V to ensure the proper conditions for deposition. During pre-electrolysis, the electrochemical environment is established, this step ensuring that the electrode surface is clean and any initial reactions that may occur at the electrodes, such as the formation of passivating films or the elimination of impurities, are completed. This process helps in stabilizing the electrolyte and electrode interface, which is essential for controlled deposition. At the same time, as DMSO and LiNO_3_ may have a few impurities or be susceptible to partial decomposition at the electrodes, the pre-electrolysis can help in breaking down any unwanted species or decomposing byproducts from the solvent or salt that could interfere with the desired deposition process later. So, the pre-electrolysis helps in altering the composition of the electrolyte, which may need to undergo certain transformations, such as solvent breakdown or changes in ion concentration, to make the system more favorable for the subsequent deposition of the target species. For example, pre-electrolysis can help achieve the necessary ion balance (e.g., Li^+^, NO^3−^,etc.) for the proper deposition of lithium. Pre-electrolysis can also help to activate the electrode surface, ensuring it is in the correct state for the deposition of the target (Li^+^) species. This could include the removal of any oxide layers or passivating films that might appear in the electrochemical reactions necessary for deposition. We can assume that the pre-electrolysis step ensures that the system is prepared, stable, and free from contaminants that could negatively affect the deposition process, allowing for a more controlled and efficient electrochemical deposition, especially in DMSO-LiNO_3_ solution.

As higher temperatures and stirring enhance ionic conductivity and improve the mass transport of lithium ions, leading to more uniform deposition, we conducted an experiment with pre-electrolysis and stirring at 313–323 K and 3.3–3.5 V. By this, we obtained a smooth deposit (Figure 7d).

#### 3.3.4. System DMSO-BMIMTFSI-LiNO_3_

After conducting all these experiments with organic solvents, we conclude that certain additives can be used to modify the deposition behavior of lithium, and we introduced the basic DMSO-LiNO_3_ anionic liquid, BMIMTFSI [1-butyl-3-methylimidazolium bis(trifluoro-methylsulfonyl)imide]. BMIMTFSI is a room temperature ionic liquid (RTIL), which is known for its non-volatile and thermally stable properties. It can act as a co-solvent with DMSO, helping to enhance the ionic conductivity and stability of the electrolyte, and we believe it can also reduce the risk of dendrite formation during lithium deposition.

Using the electrolyte DMSO–BMIMTFSI–LiNO_3_, a black deposit was obtained at an applied potential of 5.29 V and current of 0.01 A (Figure 7e). By altering the electrodeposition conditions, deposits ranging from gray to black were produced and analyzed; the corresponding results will be discussed in the following section of the article. In this system, LiNO_3_ was shown to interact with lithium metal to form a passivating layer, which plays a crucial role in suppressing dendritic growth [15,16]. The electrolyte formulation involving BMIMTFSI and LiNO_3_ aims to mitigate this issue by stabilizing and controlling the morphology of the deposited lithium.

In summary, we assumed that lithium electrodeposition from a DMSO-BMIMTFSi-LiNO_3_ electrolyte system is a promising approach in the field of lithium deposition and energy storage, with the combination of ionic liquids as additives helping to enhance the stability and efficiency of lithium metal deposition.

The electrodeposition in DMSO-BMIMTFSI-LiNO_3_ is very slow, and careful management of the electrolyte composition and electrochemical conditions is necessary to ensure optimal performance and longevity.

Table 3 presents conclusions for the electrolysis conditions of lithium electrodeposition from different electrolytes used in this study.

### 3.4. Characterization of the Lithium Deposits

#### 3.4.1. Chemical Analysis and XRD Measurements

An ICP-OES analysis of the obtained deposits revealed the presence of lithium (Li) and copper (Cu) across all electrolytes used. Additionally, trace amounts of sulfur (S) and fluorine (F) were detected in some samples, likely originating from residual electrolytes adhering to the deposits.

**Figure 9:**
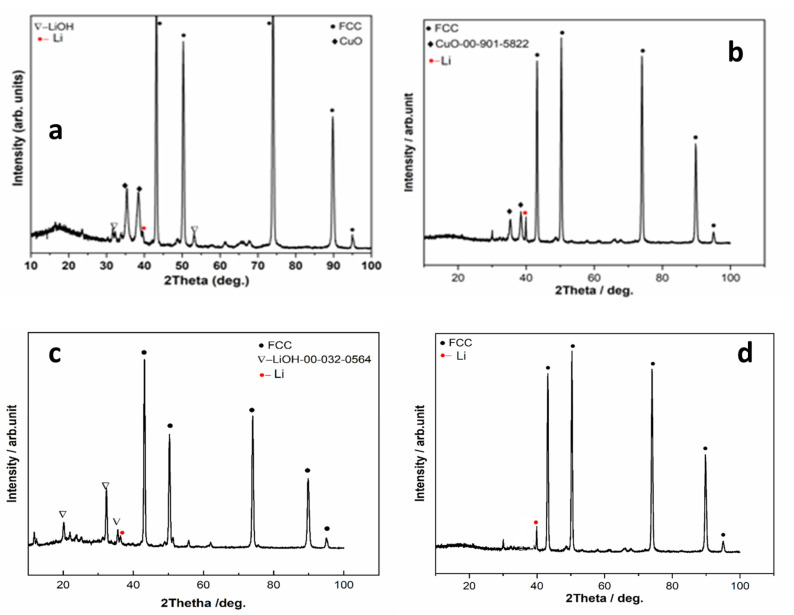
XRD patterns for the deposits obtained from the following electrolytes containing LiNO_3_ and (**a**) DMF; (**b**) DMA; (**c**) DMSO; (**d**) BMIMTFSI.

The XRD patterns of the all investigated samples show the diffraction lines, marked by the black circle at approximately 2θ values of 43.28°, 50.33°, 74.09°, 89.79°, and 95.03°, which can be indexed to the (111), (200), (220), (311), and (222) planes of the face-centered cubic (FCC) structure of copper substrates. In some samples, in addition to the diffraction lines corresponding to the FCC structure, diffraction lines associated with LiOH [17] are also observed (Figure 9a,c). The presence of LiOH is likely due to oxidation, which may result from residual water in solvents such as DMA, DMSO, or even in the electrolytes. It may also occur during sample handling in the XRD measurement process. Diffraction lines corresponding to CuO [18] originating from the substrate are observed, indicating that the deposited films are relatively thin (Figure 9a,b).

Additionally, the diffraction patterns of all the studied deposit samples exhibit diffraction lines corresponding to Li [19,20] (Figure 9).

The best lithium results were obtained for the electrolyte DMSO-BMIMTFSI-LiNO_3_ (Figure 9d). The same XRD pattern was obtained for the deposit from DMSO-LiNO_3_, corresponding to the gray deposit from Figure 7d.

#### 3.4.2. SEM Analysis

**Figure 10:**
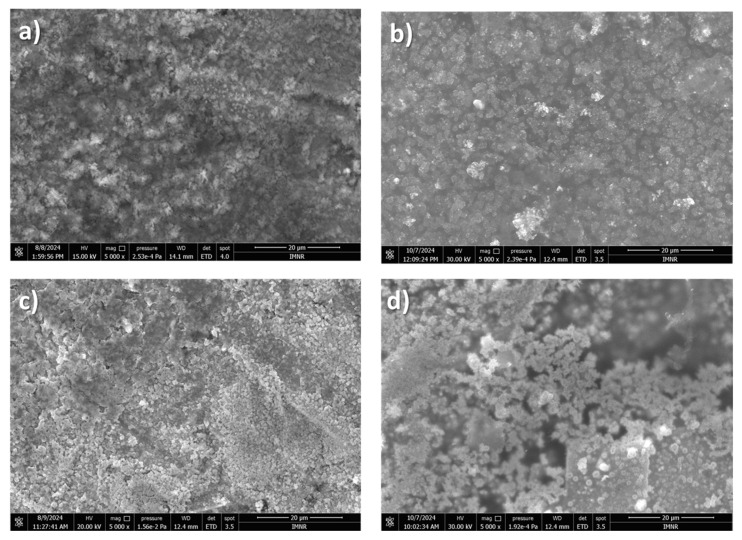
SEM images of lithium deposit from (**a**) DMF-LiNO_3_; (**b**) DMA-LiNO_3_; (**c**) DMSO-LiNO_3_; (**d**) DMSO-BMIMTFSI-LiNO_3_.

The SEM image of the lithium deposit obtained from DMF-LiNO_3_ (Figure 10a) presents a granular deposit. The granules are irregular in shape and have micrometric and submicrometric dimensions. The SEM image appears relatively coarse and irregular, with some agglomerates. This suggests non-uniform lithium plating. DMF may not be suppressing dendritic or mossy lithium growth effectively.

A semi-quantitative point chemical analysis, EDS, revealed the presence of the following elements: Cu, O, and in some areas, F and C.

The deposit obtained from DMA-LiNO_3_ (Figure 10b) shows a granular deposit, but a more compact and smoother surface than DMF. The granules have micrometric dimensions and are irregular in shape, which indicates improved lithium deposition uniformity. DMA seems to support a denser, more stable deposit, reducing the risk of dendrite formation. A semi-quantitative point chemical analysis, EDS, revealed the presence of the following elements: Cu, O, and in some areas, C.

The SEM image of the sample obtained from DMSO-LiNO_3_ (Figure 10c) presents a granular, porous, finer deposit in the interface area. Because the surface is highly porous or fibrous, possibly mossy or dendritic, the granules have micrometric and submicrometric dimensions and are irregular in shape. This suggests poor lithium deposition behavior with potential dendrite growth. DMSO might encourage faster ion transport but compromises deposit stability. A semi-quantitative point chemical analysis, EDS, revealed the presence of the following elements: Cu, S, O, and N (in some areas).

The sample from Figure 10d, which is the lithium deposit from DMSO-BMIMTFSi-LiNO_3_, presents a very low granular deposit. It appears very smooth, dense, and uniform. This is the most stable and desirable morphology. The ionic liquid additive (BMIMTFSi) likely helps form a stable solid electrolyte interphase (SEI), suppressing dendrite formation and enabling safe lithium cycling. A semi-quantitative point chemical analysis, EDS, revealed the presence of the following elements: Cu, S, O, and in some areas, F and C.

It is important to emphasize that our study generated thin lithium coatings on copper support, without dendritic shapes and of micrometric and submicrometric dimensions.

Taking into account the results obtained, we intend to develop other organic /IL electrolytes in the future that could lead to obtaining denser and larger lithium deposits.

## 4. Conclusions

- The developed systems were organic/organic-IL with the addition of LiNO_3_ and were used for the potentiostatic electrodeposition of Li on copper substrates.
- The electrodeposition process was characterized by cyclic voltammetry, and for the studied systems, the process was found to be irreversible for DMF-LiNO_3_ and DMA-LiNO_3_; diffusion-limited irreversible for DMSO-LiNO_3_; and irreversible or quasi-reversible for DMSO-BMIMTFSI-LiNO_3._
- Cyclic voltammetry was used to prove the lithium electrodeposition in the range −3.011 → −4.495 V (*vs*. Pt). Under these conditions, pure lithium deposits without dendritic shapes and of micrometric and submicrometric dimensions could be obtained by electrodeposition.
- Especially because lithium shows the highest activity among all elements (being able to generate fire or oxidize quickly when it is removed from the glove box), it was quite a challenge to obtain thin films of lithium on the copper substrate.
- The obtained deposits were characterized by chemical analysis, XRD, and SEM. Diffraction patterns reveal the existence of Li and sometimes LiOH, which was confirmed by chemical analysis. SEM images generated thin lithium coatings on copper support, without dendritic shapes and of micrometric and submicrometric dimensions.
- Moreover, the newly developed electrolyte of organic/IL allowed the electrodeposition of Li under no special conditions and proved to be an excellent solvent for lithium nitrate and to have good electrochemical stability.

## Figures and Tables

**Table 1:** Lithium salts solubilization in different solvents at room temperature.

Solvent	Lithium Salt	Observations
DMA	Li_2_CO_3_	Totally undissolved
	LiCl	Dissolved extremely slowly
	LiNO_3_	Totally dissolved
DMF	Li_2_CO_3_	Totally undissolved
	LiCl	Undissolved
	LiNO_3_	Totally dissolved
DMSO	Li_2_CO_3_	Most undissolved
	LiCl	Totally undissolved
	LiNO_3_	Totally dissolved
DMSO-BMIMTFSI	Li_2_CO_3_	Most undissolved
	LiCl	Most undissolved
	LiNO_3_	Totally dissolved

**Table 2:** Reduction potentials of metallic lithium in different electrolytes at 0.2 V/s.

Electrolyte	E_dep_^Li^ vs. QRE^Pt^ (V)
DMA + LiNO_3_	−3.815
DMF + LiNO_3_	−3.011
DMSO + LiNO_3_	−4.266
DMSO + BMIMTFSI + LiNO_3_	−4.495

**Table 3:** Conditions for the electrodeposition of lithium from different electrolytes.

Solvent	Lithium Salt	Electrodeposition Conditions
DMA	LiNO_3_	4.2 V; 0.01 A; 298 K
DMF	LiNO_3_	4 V; 0.01 A; 298 K
DMSO	LiNO_3_	5.06 V; 0.09 A; 313–323 K; stirring
DMSO + BMIMTFSI	LiNO_3_	5.29 V; 0.01 A; 313 K, stirring

## Data Availability

The original contributions presented in this study are included in the article. Further inquiries can be directed to the corresponding author.

## References

[1] Fulton L., Schäfer A.W., Sperling D. (2024). Transitions to deeply decarbonized transportation and energy systems around the world: Challenges and solutions. Transp. Res. Part. D Transp. Environ..

[2] Kasri M.A., Halizan M.Z.M., Harun I., Bahrudin F.I., Daud N., Aizamddin M.F., Shaffee S.N.A., Abd Rahman N., Shafiee S.A., Mahat M.M. (2024). Addressing preliminary challenges in upscaling the recovery of lithium from spent lithium-ion batteries by the electrochemical method: A review. RSC Adv..

[3] Xiong Y., Zhou J., Lu P., Yin J., Wang Y., Fan Z. (2022). Electrochemical lithium extraction from aqueous sources. Matter.

[4] Chen R. (2023). Ionic liquids-mediate recovery of metals from spent batteries. J. Ion. Liq..

[5] Tang W., Ma J., Zhang X., Li Y., Meng S., Zhang Y., Dong H., Liu R., Gao R., Feng M. (2024). Interfacial strategies towards highly stable Li-metal anode of liquid-based Li-metal batteries. Energy Storage Mater..

[6] Qian J., Xu W., Bhattacharya P., Engelhard M., Henderson W.A., Zhang Y., Zhang J.-G. (2015). Dendrite-free Li deposition using trace amounts of water as an electrolyte additive. Nano Energy.

[7] Wang Z., Wang H., Qi S., Wu D., Huang J., Li X., Wang C., Ma J. (2022). Structural regulation chemistry of lithium-ion solvation for lithium batteries. EcoMat.

[8] Takei T. (1979). Electrolytic deposition of lithium from non-aqueous solutions. J. Appl. Electrochem..

[9] Lin X., Khosravinia K., Hu X., Li J., Lu W. (2021). Lithium Plating Mechanism, Detection, and Mitigation in Lithium-Ion Batteries. Progress. Energy Combust. Sci..

[10] Yamada H., Yoshi K., Asahi M., Chiku M., Kitazumi Y. (2022). Cyclic Voltametry-Part.1 Fundamentals. Electrochemistry.

[11] Krtil P., Kavan L., Hoskovcová I., Kratochvilová K. (1996). Anodic oxidation of dimethyl sulfoxide based electrolyte solutions: An*in situ* FTIR study. J. Appl. Electrochem..

[12] Infrared Spectroscopy: A Quick Primer on Interpreting Spectra. https://www.masterorganicchemistry.com/2016/11/23/quick_analysis_of_ir_spectra/.

[13] Jascindra R., Hills A.E., Cerasoli E., Rakowska P.D., Ryadnov M.G. (2011). FTIR markers of methionine oxidation for early detection of oxidized protein therapeutics. Eur. Biophys. J..

[14] Mozhshukhina N., Mendez de Leo L.P., Calvo E.J. (2013). Infrared Spectroscopy Studies on Stability of Dimethyl Sulfoxide for Application in a Li–Air Battery. J. Phys. Chem. C.

[15] Levi D., Gamolsky M.D., Markovsky K., Salitra B., Levi G., Heider E., Oesten U., Schmidt R. (1999). Capacity fading of LiCoO_2_ cathodes in lithium-ion batteries: The effect of cycling, storage, temperature, and surface film formation. J. Power Sources.

[16] Qian J., Henderson W.A., Xu W., Bhattacharya P., Engelhard M., Borodin O., Zhang J.G. (2015). High rate and stable cycling of lithium metal anode. Nat. Commun..

[17] National Bureau of Standards Monograph 25, Section 17. 1980, p. 46. https://nvlpubs.nist.gov/nistpubs/Legacy/MONO/nbsmonograph25-17.pdf.

[18] Rutt O.J., Williams G.R., Simon C. (2006). Reversible lithium insertion and copper extrusion in layered oxysulfides. Chem. Commun..

[19] Covington E.J., Montgomery D.J. (1957). Sample: At T = 20 °C Note: Li6 isotope. J. Chem. Phys..

[20] Olinger B., Shaner J.W. (1983). Lithium, compression and high-pressure structure. Science.

